# Mutation of lysine 396 of the fusion protein reduces its binding affinity with integrin αVβ1 and leads to attenuation of avian metapneumovirus subtype B

**DOI:** 10.1128/mbio.00424-26

**Published:** 2026-04-07

**Authors:** Lingzhai Meng, Yuntong Chen, Mengmeng Yu, Yongzhen Liu, Suyan Wang, Xiaoxiao Xue, Wenrui Fan, Ying Wang, Tao Zhang, Peidong Guo, Ru Guo, Mingxue Hu, Xiaole Qi, Yanping Zhang, Yulu Duan, Hongyu Cui, Yulong Gao

**Affiliations:** 1 Avian Immunosuppressive Diseases Division, State Key Laboratory of Animal Disease Control and Prevention, Harbin Veterinary Research Institute, The Chinese Academy of Agricultural Sciences 111613 , Harbin, China; Charite-Universitatsmedizin Berlin, Berlin, Germany

**Keywords:** avian metapneumovirus subtype B, virulence, attenuation mechanism, αVβ1 integrin, binding

## Abstract

Avian metapneumovirus subtype B (aMPV/B) infections significantly affect the global poultry industry. However, the virulence determinants and attenuation mechanism remain unknown. Here, a series of chimeric and mutant viruses was constructed, and their pathogenicity was evaluated in a specific-pathogen-free (SPF) chicken model. First, substitutions in different genes (N, P, F, SH, G, and L) and amino acid sites (323rd, 396th, and 522nd residues in F protein) in virulent (LN16-V) and attenuated (LN16-A) viruses revealed that the residue 396th in F protein is closely related to replication ability *in vitro* and *in vivo* and is a critical determinant of aMPV/B virulence in chickens. Further studies revealed that the K396R mutation in the F protein decreases the adsorption of aMPV/B to DF-1 cells, which reduces the binding of the F protein with αVβ1 integrin. Structural and surface plasmon resonance analysis indicated that the K396R mutation primarily reduced the electrostatic potential of the RDD motif of the F protein that binds with αVβ1 and decreased the binding between the F protein and αVβ1, which is a critical determinant of the replication ability of aMPV/B. Collectively, these findings not only contribute to a better understanding of the aMPV attenuation mechanism but also offer novel strategies for developing *Metapneumovirus* members live vaccines.

**IMPORTANCE:**

Both aMPV and hMPV belong to the *Metapneumovirus* family and cause the most acute respiratory diseases in poultry and humans, respectively. Recently, an outbreak of severe respiratory disease occurred on turkey and chicken farms across different states in the USA, largely attributed to aMPV/B infections. Live-attenuated vaccines developed by the blind passage of virulent strains in tissue culture have been widely used to prevent aMPV/B infection. However, the mechanism of aMPV/B attenuation remains unclear. Here, we identified the F gene as a key determinant of the virulence of aMPV/B and confirmed that residue 396 in the F protein plays an important role in attenuating the virulence of aMPV/B. Importantly, we found that the K396R mutation decreased the binding affinity between the F protein and αVβ1 and reduced the replication ability of aMPV/B. This is the first study to identify the key virulence genes and amino acid residues of aMPV/B and elucidate the molecular mechanisms underlying the attenuation of virulence. Our work provides fundamental insights into aMPV/B pathogenicity and offers direction for guiding the rational design of novel and more effective vaccines against aMPV/B and, by extension, related pathogens, such as hMPV.

## INTRODUCTION

Avian metapneumovirus (aMPV) is an economically important pathogen causing turkey rhinotracheitis and swollen head syndrome in chickens (1, 2). Poultry disease caused by aMPV was first described in 1978, and the virus was isolated and identified in 1986 (3). Based on the level of genetic variations and antigenic differences, aMPV can be divided into four subtypes: A, B, C, and D (4, 5). Subtype B is the most common and has been detected in Asia, Africa, Europe, and South America (6). Studies conducted in Korea (7), China (8), Iraq (9), Brazil (10), Tunisia (11), and Colombia (12) reported the prevalence of aMPV/B in commercial poultry farms and live poultry markets from 2019 to 2024. Remarkably, in the USA, starting in the fall season of 2024, the spread of aMPV/B has been reported in Virginia, with subtype B being dominant in the eastern states (13). The rapid reemergence of aMPV/B in the USA is interesting because aMPV has not been reported in the USA for an extended period since the early 2000s.

aMPV is a non-segmented negative-sense RNA virus, belonging to the genus *Metapneumovirus* in the subfamily *Pneumovirinae* of the family *Paramyxoviridae* (14, 15). The viral genome consists of eight genes that encode nine proteins, including nucleocapsid (N), phosphoprotein (P), matrix protein (M), fusion protein (F), matrix protein (M2-1 and M2-2), small hydrophobic protein (SH), glycoprotein (G), and large RNA-dependent RNA polymerase (L) (16, 17). The entry of paramyxoviruses into host cells initially requires receptor recognition and fusion between the viral and cellular membranes (18–20), which is usually mediated by membrane fusion-promoting fusion proteins (F) on the viral vesicle membranes and attachment proteins designated as H, HN, or G (21). However, recombinant aMPV and human metapneumovirus (hMPV) lacking G can replicate both *in vivo* and *in vitro* (22, 23). Therefore, the F protein can mediate both attachment and membrane fusion for members of the *Pneumovirinae* subfamily, suggesting that it binds to specific cell surface receptors during attachment, drives fusion, and mediates viral entry.

Members of the genus *Parapneumovirus* F are type I integral membrane proteins that exist as inactive F0 precursors when initially folded (24). Later, F0 is cleaved into two disulfide-linked subunits, F1 and F2 (25). The F2 subunit plays a key role in stabilizing the pre-fused state of F proteins, whereas the F1 subunit is primarily responsible for receptor recognition (26). The F1 subunit contains certain regions of known function, including an N-terminal fusion peptide domain that is inserted into the target cell membrane during fusion (27), a C-terminal transmembrane anchor, and two heptad repeats, A and B (28). The large intervening region between the heptad repeats of F1 was designated as the region 2 domain (29). This domain contains regions 2a and 2b, of which the region 2a domain contains the integrin receptor-binding motif, RDD, RGD, or RSD, a key motif for receptor binding (24, 30). Integrins are a family of cell surface heterodimeric glycoproteins composed of 18 α and 8 β subunits that play pivotal roles in the adsorption and entry of a variety of viruses, including several nonenveloped viruses (such as rotavirus [31], foot-and-mouth disease virus [32], and human adenovirus [33]) and enveloped viruses (Epstein-Barr virus [34], Sindbis virus [35], and yellow fever virus [36]). Among them, αVβ1 is primarily responsible for binding to the RDD motif of F proteins, thereby facilitating aMPV/B adsorption and entry.

Previous studies have shown that serial *in vitro* passages can attenuate viral pathogenicity in animals, and several aMPVs have been successfully attenuated by serial *in vitro* passages (37). The aMPV/C MN/turkey/1-a/97 strain was shown to be sufficiently attenuated after 63 serial passages in cell culture (38). Turkeys inoculated with the aMPV/A 8544 strain passaged for 18–22 generations in chicken embryo tracheal organ cultures via the eye drop route did not show any clinical signs (39). Furthermore, a serial passages study of aMPV/B showed that the VCO3/60616 strain (turkey-derived) was completely attenuated after 50 passages *in vitro* and was used for the production of live-attenuated vaccines (40). In our previous study, an attenuated LN16-A strain was developed by serial passaging of Vero cells. Genomic differences between LN16-A and the parental LN16-V virus were predominantly concentrated in the N, P, F, SH, G, and L genes (8, 41). However, the mechanisms underlying the attenuation of aMPV remain unclear.

Identification of virulence genes and amino acid sites of aMPV is essential for the rapid development of novel vaccines and delineation of attenuation mechanisms (42, 43). Therefore, in this study, we aimed to identify the molecular determinants responsible for the differences in virulence observed between strains LN16-V and LN16-A. We found that the F gene is a critical virulence determinant for aMPV/B and that the residue at position 396 of the F protein is the molecular determinant of this virulence. Furthermore, the mechanisms underlying aMPV/B attenuation of the LN16-A virulence in chickens were investigated. Our findings will provide a better understanding of the key molecular mechanisms underlying aMPV attenuation, with implications for guiding the rational design of novel and more effective vaccines.

## MATERIALS AND METHODS

### Animals

Specific pathogen-free (SPF) white leghorn chickens were obtained from the Animal Experiment Center of the Harbin Veterinary Research Institute (HVRI), Chinese Academy of Agricultural Sciences. Blood samples were evaluated using avian rhinotracheitis antibody test kits (IDEXX Laboratories Inc., Westbrook, MA, USA), which confirmed that all SPF chickens were free of aMPV infection.

### Cells and viruses

Vero, DF-1, 293T, and BSR-T7/5 cells (expressing T7 RNA polymerase), stored at our laboratory (44, 45), were cultured in Dulbecco’s modified Eagle’s medium (DMEM, D6429, Gibco Life Technologies, Shanghai, China) supplemented with 10% fetal bovine serum (FBS, 10099-141, Gibco) and antibiotics (100 U/mL penicillin and 100 µg/mL streptomycin). 293F cells were maintained in serum-free DMEM (H310KJ, BasalMedia) in the presence of 5% CO_2_ at 37°C. The virulent aMPV/B strain, LN16-V (GenBank: MH745147), was isolated, identified, and preserved in our laboratory (8). The attenuated strain, LN16-A (GenBank: PP069785), was derived by serial passaging of Vero cells for 30 passages.

### Plasmids

Using a reverse genetics system established in our laboratory (46), full-length cDNA plasmids of the virulent LN16-V and attenuated rLN16-A strains (pOK-LN16V and pOK-LN16A) were constructed. Following transfection, we obtained the rescued viruses, rLN16-V and rLN16-A. Plasmids expressing chicken integrin receptors, ch-αV-HA and ch-β1-HA (with an HA tag for C-terminal fusion), were preserved in our laboratory (47). Soluble eukaryotic expression plasmids expressing chicken integrin receptor proteins (pCAG-αV-His and pCAG-β1-His) and eukaryotic expression plasmids expressing the F1 domain of the fusion protein (pCAG-rLN16VF1-His and pCAG-rK396RF1-His) were synthesized by Invitrogen (Shanghai, China). These plasmids contained a signal peptide (48) at the N-terminus and a His tag at the C-terminus.

### Construction of full-length cDNAs for chimeric and site-mutated viruses

Using the primers listed in Table 1 (pN-F/R, pP-F/R, pF-F/R, pSH-F/R, pG-F/R, and pL-F/R), the full-length cDNA plasmids, pOK-LN16V and pOK-LN16A, were subjected to inverse polymerase chain reaction (PCR) to generate 12 linearized vectors with gene substitutions. The PCR procedure is 94°C for 1 min, followed by 30 cycles of 98°C for 10 s, 55°C for 15 s, and 68°C for 4 min. Six gene (N, P, F, SH, G, and L) fragments of the LN16-V and LN16-A strains were amplified using PCR with the cDNAs of virulent LN16-V and attenuated LN16-A as templates (Table 1), and a total of 12 gene fragments were obtained. Following the instructions of the ClonExpress II one-step cloning kit (C112, Vazyme), the 12 linearized vectors and gene fragments were recombined to obtain 12 full-length cDNA infectious clone plasmids with the following gene substitutions: pOKLN16-VAN, pOKLN16-VAP, pOKLN16-VAF, pOKLN16-VASH, pOKLN16-VAG, pOKLN16-VAL, pOKLN16-AVN, pOKLN16-AVP, pOKLN16-AVF, pOKLN16-AVSH, pOKLN16-AVG, and pOKLN16-AVL, respectively. Subsequently, using pOKLN16-VAF and pOKLN16-AVF as templates, amino acids 323, 396, and 522 of the F protein were sequentially mutated using the Mut Express II rapid mutagenesis kit (C214, Vazyme). Six full-length cDNA infectious clone plasmids with F protein site mutations (pOKLN16-E323K, pOKLN16-K396R, pOKLN16-L522P, pOKLN16-K323E, pOKLN16-R396K, and pOKLN16-P522L) were obtained.

**TABLE 1:** Primers for the construction of gene chimeric viruses

Name	Primer sequence (5’−3’)
pN-F	CTGTTAGCACAGTGTCTGAAAAAGTTCCA
pN-R	AGCCTCTCTCTCATCTTCAGTTAG
pP-F	TGGAACCACCAGAGAACTAGGTATGTCC
pP-R	TGGATCTTCTCACAGAGGTGGAATTCTTG
pF-F	GTCCAGAAGGAATCCCTGCAGATATGAA
pF-R	ATTCTTCACTGTAAGTTTCTTGTATCTTCC
pSH-F	ACGGGACAAGTATCCAGATGGGGTCAGA
pSH-R	TGGCTAATGCAGCACCTATGTTACTCTTG
pG-F	CTTGCATTATAGGTCGACCATACCTAAAG
pG-R	ATGGGTTCACCCCTTCTCTAATTGTAAC
pL-F	ATTTCCACTTTGACACAAGCCCTATCAA
pL-R	TATGACTCTCTATGGCAGTGTTAGAAATT
N-F	CAACAGAAAAGAAAAATGGGACAAGTAAA
N-R	TCAGACACTGTGCTAACAGGGTCA
P-F	ACCTCTGTGAGAAGATCCATTAGTG
P-R	TAGTTCTCTGGTGGTTCCATGTTT
F-F	GAAACTTACAGTGAAGAATCATGCAG
F-R	GCAGGGATTCCTTCTGGACATCTTT
SH-F	CATAGGTGCTGCATTAGCCATC
SH-R	ATCTGGATACTTGTCCCGTCTTC
G-F	AGAGAAGGGGTGAACCCATC
G-R	GGTCGACCTATAATGCAAGACC
L-F	ACTGCCATAGAGAGTCATAGGTATC
L-R	CTTGTGTCAAAGTGGAAATTAGTTG

### Virus rescue

As described previously (46), full-length cDNA plasmids were co-transfected with the supporting plasmids, pCAGGS-N, pCAGGS-P, pCAGGS-M21, and pCAGGS-L, into BSR-T7/5 cells, with untransfected BSR-T7/5 cells acting as negative controls. After 96 h, the transfected BSR-T7/5 cells and supernatants were subjected to two freeze-thaw cycles and subsequently inoculated into Vero cells. The cytopathic effects were monitored daily. Genomic RNA from each rescued virus was extracted and subjected to reverse transcription (RT)-PCR to confirm successful rescue of all viruses.

### Indirect immunofluorescent assay (IFA)

Vero cells were infected with the virus at a multiplicity of infection (MOI) of 0.01. After incubation at 37°C for 1 h, the viral inoculum was removed, and the cells were washed thrice with PBS. Each rescued virus was identified using IFA, as described previously (46).

### Western blotting

DF-1 cells were infected with the viruses at MOI of 0.01, and the expression of N proteins was detected via western blotting at different time points. Samples were separated using 12% sodium dodecyl sulfate-polyacrylamide gel electrophoresis and transferred to nitrocellulose membranes. The membrane was blocked using QuickBlock Western (P0252, Beyotime) for 1 h at 37°C and then incubated with mouse anti-aMPV-N antibodies for 1 h at 37°C. The membranes were washed thrice with PBS containing 0.05% Tween 20 (PBST). The membranes were then incubated with IRDye 800CW goat anti-mouse IgG (926-32212, LI-COR Biosciences, NE, USA) for 1 h at room temperature. Finally, the membranes were washed thrice with PBST and analyzed using a Licor ODYSSEY instrument (Licor, LI-COR Biosciences). Gray-scale analysis of protein expression was performed using the ImageJ software (National Institutes of Health).

### Virus growth curve

To assess the growth kinetics of the rescued viruses, DF-1 cells in 6-well plates were infected with individual rescued viruses or the parental virus at an MOI of 0.01. After incubation at 37°C for 1 h, the viral inoculum was removed, and the cells were washed thrice with PBS. The cells were then incubated at 37°C in a 5% CO_2_ incubator for continued cultivation. The infected cells were harvested at 24, 48, 72, 96, and 120 h post-infection (hpi). Viral titers were determined via TCID_50_ titration of Vero cells in 96-well plates at each time point.

### Pathogenicity analysis of the rescued virus

To identify the key genes that determine the virulence of aMPV/B, 3-week-old SPF chickens (10 per group) were intranasally inoculated with 2 × 10^4^ TCID_50_ of rLN16-A, rLN16-V, or 12 chimeric viruses. Ten mock-infected chickens (healthy control) received 0.2 mL of DMEM. Chickens in each group were reared separately in different isolators and observed daily for 7 days. To further identify the key amino acid residues associated with aMPV/B virulence, six F gene site-mutated viruses (rE323K, rK396R, rL522P, rK323E, rR396K, and rP522L) were inoculated into 3-week-old SPF chickens (10 per group), and clinical symptoms were observed from 1 to 7 days post-infection (dpi). Viral shedding in each group was measured using RT-quantitative PCR (RT-qPCR), and histopathological examination was performed as described previously (49).

### Genetic stability analyses

To evaluate the genetic stability of the 396th residue in the F protein, the rK396R was passaged for five generations in both DF-1 and Vero cells. The RNA from the F1 and F5 generations of the rK396R was reverse-transcribed into cDNA using PrimeScript Strand cDNA Synthesis Kit (6210A; Takara, China). The F gene fragments from each passage were amplified by PCR with primers F-F and F-R (Table 1) and subjected to Sanger sequencing. In addition, Illumina short-read depth sequencing was performed on the whole genome of the rescued virus rK396R, fifth-generation rK396R (DF-1 cells), and rK396R infecting chickens (2, 3, and 4 dpi) as previously described (50).

### Virus-cell attachment assay

DF-1 cells were infected with rLN16-V and rK396R derived in Vero cells (equal number of genome copies) at an MOI of 50. After adsorption at 4°C for 1 or 2 h, the cells were washed thrice with PBS. Cellular RNA was extracted after infection using the TRIzol reagent (15596018, Invitrogen), and reverse transcription was performed according to the manufacturer’s instructions (K1691, Thermo Scientific). Chicken 28S RNA was used as the reference gene (51). aMPV/B mRNA levels were detected using RT-qPCR as described previously (46). Additionally, the adsorbed virus was detected using IFA with polyclonal antibodies against the aMPV/B-N protein and examined using a laser confocal microscope (LSM980, Zeiss).

### Flow cytometry

The DF-1 cells were digested with trypsin, centrifuged at 800 × *g* for 5 min, and washed thrice with ice-cold PBS. The cells were then infected with rLN16-V and rK396R at an MOI of 50 and allowed to adsorb at 4°C for 1 h, followed by three washes with ice-cold PBS. After fixing with 4% paraformaldehyde, the cells were incubated with a polyclonal antibody against the N protein of aMPV/B for 1 h and subsequently stained with fluorescein isothiocyanate (FITC)-conjugated goat anti-mouse IgG. Stained cells were analyzed using a FACS-Aria II flow cytometer (52).

### Purification and quantification of soluble proteins

The pCAG-αV-His, pCAG-β1-His, pCAG-rLN16VF1-His, and pCAG-rK396RF1-His plasmids were transfected into 293F cells using the PolyJet transfection reagent (SL100688, Signagen) for protein purification. At 96 h post-transfection, the cell culture medium was collected and purified using prepacked HisTrap Ni-Sepharose columns (17371201, Cytiva), as described previously (27). The purified proteins were quantified using a protein quantification kit (KTD3002, Abbkine).

### Binding assay

293T cells were transfected with either ch-αV-HA or ch-β1-HA. DF-1 cells were transfected with either siRNA targeting chicken integrin αV (Si-αV) or β1 (Si-β1). The sequences are listed as follows: Si-αV, sense, GGCUAAAUAUGACUCCA, antisense, UGGAGUCAUAUUUAGCC; Si-β1, sense, GCUCAGUCUUACUAGUGAA, antisense, UUCACUAGUAAGACUGAGC. After 36 h of transfection, the cells were digested with trypsin, centrifuged at 800 × *g* for 4 min, and washed thrice with ice-cold PBS. The cells were then incubated with various viruses or F1 proteins at 4°C for 1 h, followed by incubation with a 1:300 dilution of anti-aMPV-N polyclonal antibody or anti-His tag polyclonal antibody (10001-0-AP, Thermo Fisher) for 1 h. The cells were then washed thrice with ice-cold PBS. The cells were fixed using 4% paraformaldehyde (P1110, Solarbio) at 25°C for 15 min. Finally, the cells were stained with a 1:300 dilution of monoclonal anti-HA-TRITC antibody produced in mouse (H9037, Sigma) for 1 h at 4°C. After two washes, the cells were resuspended in PBS and analyzed using flow cytometry with an LSRII flow cytometer (Cytomics FC 500; BD Biosciences).

### Structure analysis

The structures of the F proteins of rLN16-V and rK396R and the chicken αVβ1 integrin were predicted using the open-source server Rosseta (53) and AlphaFold (54) based on the human metapneumovirus fusion protein (PDB ID: 5WB0) and human integrin αVβ1 (PDB ID: 7NXD), respectively. The constructed model was optimized using molecular dynamics simulations. The structures of the proteins were obtained using the PYMOL mutagenesis wizard. The initial docking model of chicken αVβ1 integrin and F proteins of rLN16-V and rK396R was performed at hdock (http://hdock.phys.hust.edu.cn/). The highest-scoring model was further optimized using Rosetta 3.12 (https://downloads.rosettacommons.org/downloads/academic/3.12/) to obtain a final docking model (https://github.com/Famiglistimo-ltm/paper-source-data.git). The binding area, binding energy, and packing density of the protein-protein interface were evaluated using the Rosetta Interface Analyzer (55). The amino acids at the protein-protein interaction interface were analyzed using the PLIP web server (56) and Ligplot (57).

### Surface plasmon resonance analysis

Sodium acetate (pH 4, 10 nM) was used as the coupling buffer. Chicken integrin αV or β1 was coupled to a Series S Sensor Chip CM5 (BR100530, Cytiva) using the amine coupling kit (BR100050, Cytiva), with a maximum coupling response of 350 RU. PBST was used as the running buffer for the immobilization, kinetic, and inhibition studies. Analytes were dissolved in running buffer, and a flow rate of 30 μL/min was used for association (180 s) and dissociation (600 s) at a constant temperature of 25°C. Glycine-HCl (pH = 1.5) was injected for 30 s at a flow rate of 30 mL/min for regeneration and to achieve a prior baseline status. The response curves of various analyte concentrations were globally fitted to a 1:1 binding model using the Biacore T200 (ACROBiosystems, China) evaluation software.

### Statistical analysis

Statistical analyses were performed using GraphPad Prism 8.0.2. Data are presented as means ± standard deviation (SD). Student’s *t*-test was used to assess differences between two groups, and two-way analysis of variance (ANOVA) was used to independently compare multiple groups for each treatment. Statistical significance was set at *P* < 0.05.

## RESULTS

### Recovery and *in vitro* growth properties of the gene chimeric viruses

**Fig 1:**
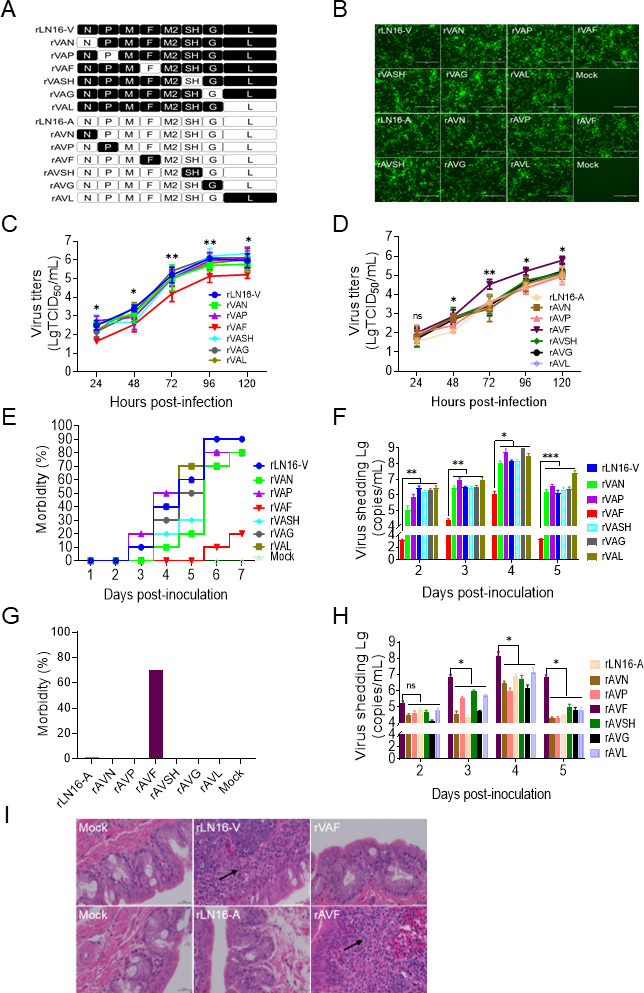
Identification of critical virulence genes of aMPV/B. (**A**) Schematic showing the construction of gene chimeric viruses. The genes of rLN16-V and rLN16-A are indicated with black and white rectangles, respectively. (**B**) IFA analysis of gene chimeric viruses. The N protein was detected in the Vero cells inoculated with the viruses using rabbit anti-aMPV-N antibody; the rLN16-V-infected Vero cells were used as the positive control; mock-infected Vero cells were used as the negative control. Scale bar, 200 μm. (**C**) Comparison of the growth kinetics of rescued viruses with rLN16-V as the backbone (rVAN, rVAP, rVAF, rVASH, rVAG, and rVAL) in DF-1 cells. DF-1 cells are incubated with rLN16-V, rVAN, rVAP, rVAF, rVASH, rVAG, and rVAL at an MOI of 0.01 and then quantified using a TCID_50_ assay at 24, 48, 72, 96, and 120 hpi. The asterisk indicates a significant difference in the viral titers between rLN16-V and rVAF. (**D**) Comparison of the growth kinetics of rescued viruses with rLN16-A as the backbone (rAVN, rAVP, rAVF, rAVSH, rAVG, and rAVL) in DF-1 cells. DF-1 cells are incubated with rLN16-A, rAVN, rAVP, rAVF, rAVSH, rAVG, and rAVL at an MOI of 0.01 and then quantified using a TCID_50_ assay at 24, 48, 72, 96, and 120 hpi. The asterisk indicates a significant difference in the viral titers between rLN16-A and rAVF. (**E**) Morbidity of SPF chickens after inoculation with rLN16-V, rVAN, rVAP, rVAF, rVASH, rVAG, and rVAL at 1-7 dpc (*n* = 10). Clinical signs were observed daily in each group (*n* = 10) from 1 to 7 days post-challenge (dpc), with morbidity confirmed based on the presence of nasal scabs or turbid nasal fluid. (**F**) Viral shedding of SPF chickens after inoculation with rLN16-V, rVAN, rVAP, rVAF, rVASH, rVAG, and rVAL. Choanal swabs were collected at 2-5 dpc, and viral shedding was quantified using RT-qPCR (*n* = 10). (**G**) Morbidity of SPF chickens after inoculation with rLN16-A, rAVN, rAVP, rAVF, rAVSH, rAVG, and rAVL at 1-7 dpc (*n* = 10). (**H**) Viral shedding of SPF chickens after inoculation with rLN16-A, rAVN, rAVP, rAVF, rAVSH, rAVG, and rAVL at 2–5 dpc (*n* = 10). (**I**) Histopathological changes in chickens of the rLN16-V, rVAF, rLN16-A, and rAVF infection groups (*n* = 3). Hematoxylin and eosin (H & E) staining of turbinates at 9 dpc. Scale bar, 200 μm. Data are shown as means ± SD for triplicates from a representative experiment. *P < 0.05; **P < 0.01; ***P < 0.001. ns, no significant difference.

Previously, we have found that the differences in the complete genome sequence of the virulent strain, LN16-V (GenBank: MH745147), and the attenuated strain, LN16-A (GenBank: PP069785), were primarily in six genes: N, P, F, SH, G, and L (41). To further identify the key virulence genes of LN16-V, a total of 12 gene chimeric viruses full-length cDNAs were constructed by sequentially exchanging six genes (N, P, F, SH, G, and L) with rLN16-V or rLN16-A as backbone (Fig. 1A). Twelve full-length cDNAs and supporting plasmids (pCAGGS-N, pCAGGS-P, pCAGGS-M21, and pCAGGS-L) were co-transfected into BSR-T7/5 cells as described previously (46). After the second passage, the cells from the 12 transfected groups developed severe cytopathic lesions, including shrinkage, detachment, and syncytial plaques. The IFA assay further confirmed that the 12 gene chimeric viruses were successfully rescued (Fig. 1B). The six-gene chimeric viruses rescued using the rLN16-V backbone were named rVAN, rVAP, rVAF, rVASH, rVAG, and rVAL, and the six chimeric viruses rescued using the rLN16-A backbone were named rAVN, rAVP, rAVF, rAVSH, rAVG, and rAVL. All 12 chimeric viruses were identified using molecular sequencing (Fig. S1).

To compare the growth properties of these chimeric viruses and their parental viruses, the growth kinetics were analyzed by infecting DF-1 cells with each virus. As shown in Fig. 1C, rVAF showed significantly lower viral titers than the rLN16-V and other chimeric strains (rVAN, VAP, VASH, VAG, and VAL) at 24–120 hpi. However, with the F gene substitution as the rLN16-A backbone, rAVF exhibited higher replication levels than rLN16-A and other chimeric strains (rAVN, AVP, AVSH, AVG, and AVL) (Fig. 1D), indicating that F gene substitution can significantly affect aMPV/B replication in DF-1 cells.

### F gene plays a critical role in determining the pathogenicity of aMPV/B

Next, the pathogenicities of the six chimeric viruses (rVAN, rVAP, rVAF, rVASH, rVAG, and rVAL) rescued with rLN16-V as the backbone, and the parental virus rLN16-V, were evaluated in 21-day-old SPF chickens at a dose of 2 × 10^4^ TCID_50_^−1^. Chickens in the rVAN, rVAP, rVASH, rVAG, rVAL, and rLN16-V inoculation groups developed a large amount of turbid nasal fluid with symptoms of head flopping and depression within 1-7 dpi, with morbidity rates of 80%, 80%, 80%, 80%, 90%, and 90%, respectively. In contrast, only two chickens in the rVAF inoculation group developed a small amount of nasal fluid, with a morbidity rate of 20% (Fig. 1E). The results of RT-qPCR showed that the viral shedding levels of rVAF-infected chickens were significantly lower than those of rLN16-V, rVAN, rVAP, rVASH, rVAG, and rVAL (Fig. 1F). These results suggested that the F gene is closely associated with aMPV/B pathogenicity.

To further assess the role of the F gene in aMPV/B pathogenicity, six chimeric viruses (rAVN, rAVP, rAVF, rAVSH, rAVG, and rAVL) rescued with rLN16-A as the backbone and the parental virus rLN16-A were inoculated into 21-day-old SPF chickens at a dose of 2 × 10^4^ TCID_50_^−1^. The results showed that chickens in the rAVN, rAVP, rAVSH, rAVG, rAVL, and parental virus rLN16-A inoculation groups did not show any clinical signs (0% morbidity rate), whereas seven chickens in the rAVF infection group developed viscous turbid nasal fluid with a morbidity rate of 70% (Fig. 1G). The results of RT-qPCR showed that the viral shedding levels of chickens in the rAVF group were significantly higher than those in the rLN16-A, rAVN, rAVP, rAVSH, rAVG, and rAVL groups (Fig. 1H). In addition, the results of histopathological analysis showed that rVAF and attenuated rLN16-A did not cause obvious histopathological lesions in the turbinates of the inoculated chickens. In contrast, chickens infected with rAVF and virulent rLN16-V showed a large amount of inflammatory cell infiltration and necrosis in the lamina propria of the posterior turbinate (Fig. 1I). Taken together, these results suggested that the F gene is critical for the difference in pathogenicity between the virulent and attenuated aMPV/B strains.

### Residue 396 plays a critical role in determining the pathogenicity of aMPV/B

**Fig 2:**
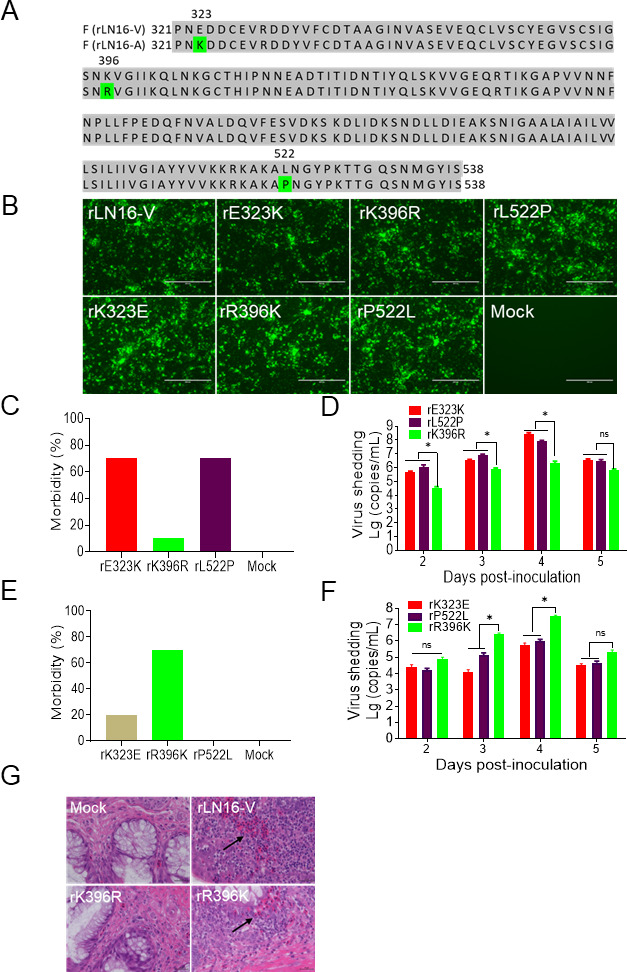
Identification of critical virulence amino acid sites of the F protein in aMPV/B. (**A**) Analysis of the differences in the sequences of the F proteins (321–538 amino acids) of rLN16-V and rLN16-A. Differences in amino acid sequence at sites 323, 396, and 522 of the F protein. (**B**) IFA analysis of the F protein single amino acid-mutated viruses. The N protein was detected in the Vero cells inoculated with the rE323K, rK396R, rL522P, rK323E, rR396K, and rP522L using a rabbit anti-aMPV-N antibody; the rLN16-V strain-infected Vero cells were used as the positive control; mock-infected Vero cells were used as the negative control. Scale bar, 200 μm. (**C**) Morbidity of SPF chickens after inoculation with rE323K, rK396R, and rL522P. Clinical signs were observed daily in each group (*n* = 10) from 1 to 7 dpc, with morbidity confirmed based on the presence of nasal scabs or turbid nasal fluid. (**D**) Viral shedding of SPF chickens after inoculation with rE323K, rK396R, and rL522P. Choanal swabs were collected at 2–5 dpc, and viral shedding was quantified using RT-qPCR (*n* = 10). (**E**) Morbidity of SPF chickens after inoculation with rK323E, rR396K, and rP522L at 1-7 dpc (*n* = 10). (**F**) Viral shedding of SPF chickens after inoculation with rK323E, rR396K, and rP522L at 2–5 dpc (*n* = 10). (**G**) Histopathological changes in chickens of the rLN16-V, rK396R, and rR396K infection groups (*n* = 3). H & E staining of turbinates at 9 dpc. Scale bar, 200 μm. Data are shown as means ± SD for triplicates from a representative experiment. *P < 0.05; ns, no significant difference.

Sequence analysis indicated a difference in three amino acids (sites 323, 396, and 522) in the F protein between LN16-V and LN16-A (Fig. 2A). To assess the contribution of individual amino acids in the F protein to aMPV/B pathogenicity, F gene single amino acid mutant viruses with the rLN16-V backbone (rE323K, rK396R, and rL522P) and rLN16-A backbone (rK323E, rR396K, and rP522L) were rescued. All six F gene single amino acid mutant viruses were identified using molecular sequencing (Fig. S2). The IFA confirmed that the six F gene single amino acid mutant viruses were successfully rescued (Fig. 2B). Subsequently, the pathogenicity of the rE323K, rK396R, and rL522P was evaluated by inoculating 21-day-old SPF chickens at a dose of 2 × 10^4^ TCID_50_^−1^. The results showed that chickens in the rE323K and rL522P inoculation groups developed a large amount of nasal fluid (70% morbidity rate) within 1–7 dpi, whereas only one chicken in the rK396R inoculation group developed a small amount of nasal fluid, with a morbidity rate of 10% (Fig. 2C). The results of RT-qPCR showed that the viral shedding levels in the rK396R inoculation group were significantly lower than those in the rE323K and rL522P groups (Fig. 2D). These results suggested that the attenuation of LN16-A results from a lysine (K)-to-arginine (R) mutation at amino acid 396 (K396R) in the F protein. To further evaluate the role of amino acid 396 of the F protein in aMPV/B pathogenicity, 21-day-old SPF chickens were inoculated with rK323E, rR396K, or rP522L at a dose of 2 × 10^4^ TCID_50_^−1^. Seven chickens in the rR396K inoculation group showed a large amount of turbid nasal fluid and a morbidity rate of 70%, which was significantly higher than those in the rK323E (20%) and rP522L (0%) inoculation groups (Fig. 2E). Correspondingly, the viral shedding levels in the rK323E and rP522L inoculation groups were significantly lower than those in the rR396K group (Fig. 2F).

In addition, the results of histopathological analysis showed that chickens in the rK396R inoculation group did not develop any obvious pathological tissue damage, whereas those in the rR396K inoculation group developed numerous inflammatory cell infiltrates and local hemorrhages in the lamina propria of the nasal turbinate (Fig. 2G). These results clearly indicated that amino acid 396 of the F protein is the key virulence site that determines the pathogenicity of aMPV/B.

### K396R mutation in the F gene decreases the replication ability of aMPV/B *in vitro*

**Fig 3:**
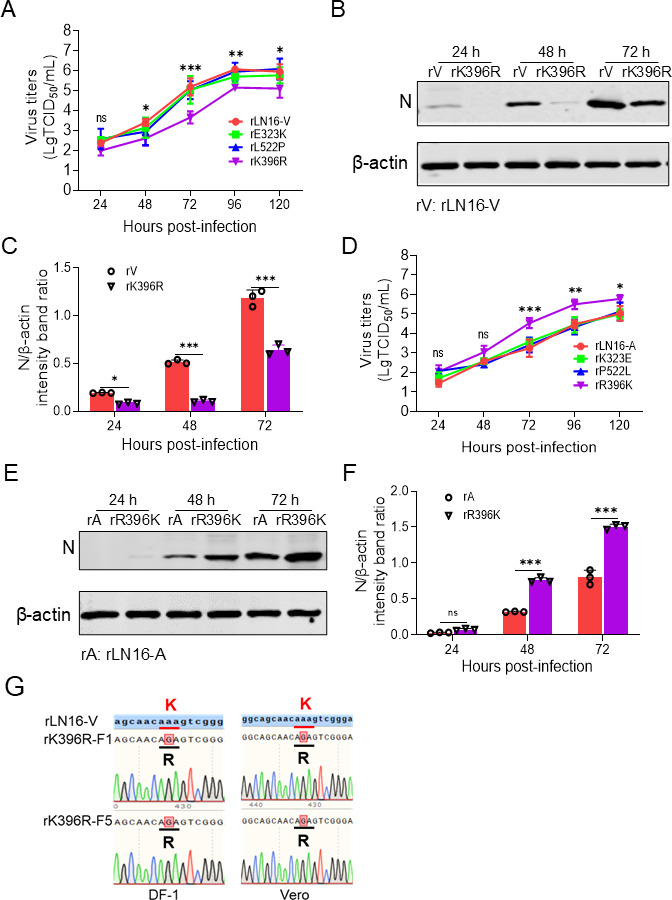
Effect of mutating residue 396 of the F protein of aMPV/B on viral replication. (**A**) Growth kinetics of the rE323K, rK396R, and rL522P in DF-1 cells. DF-1 cells were infected with different viruses at an MOI of 0.01, which were harvested and quantified using a TCID_50_ assay at 24, 48, 72, 96, and 120 hpi. The asterisk indicates a significant difference in the viral titers between rLN16-V and rK396R. (**B**) Comparison of rLN16-V and rK396R N protein expression levels. DF-1 cells were infected with rLN16-V and rK396R at an MOI of 0.01, and N protein expression was detected by Western blotting at 24, 48, and 72 hpi. β-actin expression in the cell lysates was used as the internal control. (**C**) The N/β-actin intensity ratio was normalized to the control. DF-1 cells were inoculated with rLN16-V and rK396R at an MOI of 0.01, and the relative ratio of N/β-actin was detected. (**D**) Growth kinetics of the rK323E, rR396K, and rP522L in DF-1 cells. DF-1 cells were infected with different viruses at an MOI of 0.01, which were harvested and quantified using a TCID_50_ assay at 24, 48, 72, 96, and 120 hpi. The asterisk indicates a significant difference in the viral titers between rLN16-A and rR396K. (**E**) Comparison of rLN16-A and rR396K N protein expression levels. DF-1 cells were infected with rLN16-A and rR396K at an MOI of 0.01, and N protein expression was detected by western blotting at 24, 48, and 72 hpi. β-actin expression in the cell lysates was used as the internal control. (**F**) The N/β-actin intensity ratio was normalized to the control. DF-1 cells were inoculated with rLN16-A and rR396K at an MOI of 0.01, and the relative ratio of N/β-actin was detected. (**G**) Sanger sequencing results for the 396th residue in the F protein of different generations of rK396R. The rK396R was passaged five generations serially in DF-1 and in Vero cells, followed by sequencing of the 396th residue in the F protein. Data are shown as means ± SD for triplicates from a representative experiment. *P < 0.05; **P < 0.01; ***P < 0.001; ns, no significant difference.

Viral pathogenicity and virulence are usually closely related to its replication ability (43, 58, 59). To further assess the role of the F protein residue 396 in viral replication, the growth curves of the F gene single amino acid mutant viruses, rK396R and rR396K, were compared with those of the respective parental viruses by infecting DF-1 cells. The results showed that the replication level of rK396R in DF-1 cells was significantly lower (approximately 6-fold to 12-fold) than that of rLN16-V, rE323K, and rL522P (Fig. 3A). Western blotting showed that the expression of the N protein was significantly lower in rK396R than in rLN16-V at 24–72 hpi (Fig. 3B and C). Furthermore, after mutating R396 of the F gene to K, the replication level of rR396K in DF-1 cells was significantly higher (approximately 2.5-fold to 8-fold) than those of rLN16-A, rK323E, and rP522L (Fig. 3D). Western blotting showed that the expression level of the N protein of rR396K was significantly higher than that of the attenuated rLN16-A at 24–72 hpi (Fig. 3E and F). These results suggested that the K396R mutation in the F gene reduced the efficiency of aMPV/B replication in DF-1 cells.

To further validate whether reversion mutation occurs at residue 396 of the F protein, rK396R was passaged for five generations in DF-1 and Vero cells. The results of Sanger sequencing indicate that the 396th residue of the F protein from various generations remains R, with no evidence of reversion (Fig. 3G). In addition, the results of Illumina short-read depth sequencing revealed that in the rK396R, fifth-generation rK396R (DF-1 cells), and rK396R infecting chickens (2, 3, and 4 dpi), the 396th residue of the F protein consistently retained R. The sequencing coverage was 98.93%, with an average depth of 338,850.50, a site error model of 0.02%, and a variant frequency of 0.0074%. Collectively, these results clearly indicate that rK396R has high genetic stability during both *in vivo* and *in vitro* passages.

### The K396R mutation decreased the adsorption of aMPV/B to DF-1 cells

**Fig 4:**
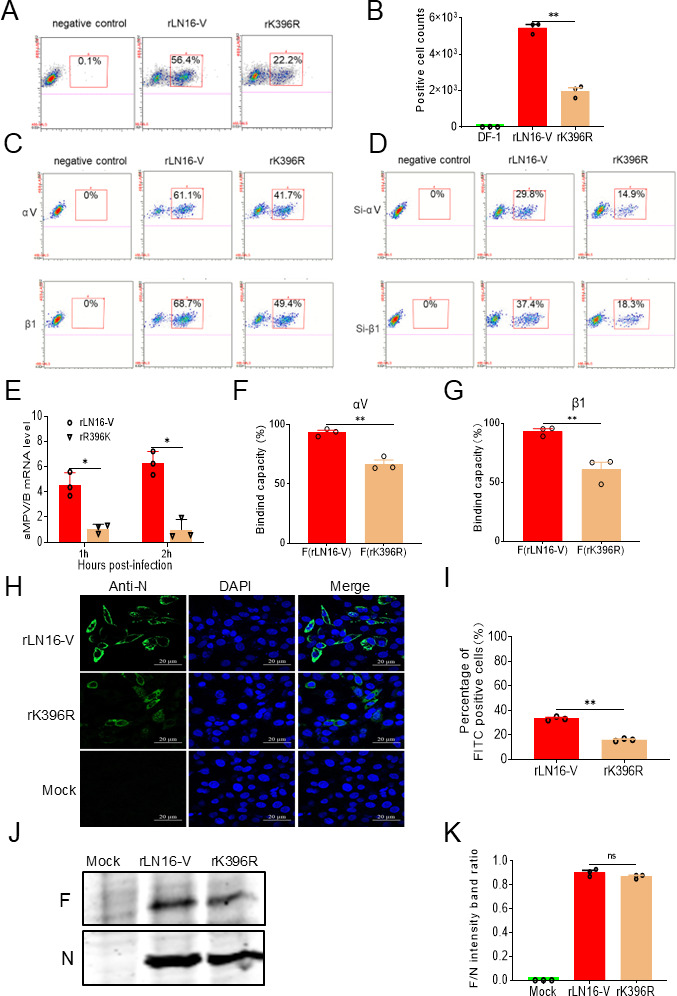
Detecting the adsorption of rLN16-V and rK396R on the DF-1 cells. (**A**) Flow cytometry analysis of the percentage of FITC-positive cells in the rLN16-V and rK396R infection groups. DF-1 cells were infected with rLN16-V and rK396R at an MOI of 50 for 1 h at 4°C. The virus-infected cells were incubated with a polyclonal antibody against the N protein of aMPV/B for 1 h and subsequently stained with FITC-conjugated goat anti-mouse IgG for 1 h. (**B**) The mean average values of FITC-positive cells (10,000 events captured per sample) were assessed using a FACS-Aria II flow cytometer. The results are shown as the average of the number of FITC-positive cells in three independent experiments. (**C**) Flow cytometry analysis of the percentage of FITC-positive cells after overexpressing ch-αV-HA or ch-β1-HA. Following transfection of 293T cells with either ch-αV-HA or ch-β1-HA, rLN16-V, and rK396R were respectively infected at an MOI of 50 at 4°C. The virus-infected cells were incubated for 1 h with polyclonal anti-aMPV/B N protein antibody, followed by staining with FITC-conjugated goat antisera against mouse IgG and mouse anti-HA-TRITC monoclonal antibody (H9037, Sigma) at 4°C for 1 h. Flow cytometric analysis was performed using an LSRII flow cytometer. (**D**) Flow cytometry analysis of the proportion of FITC-positive cells following interference with Si-αV or Si-β1. Following transfection of DF-1 cells with si-αV or si-β1, rLN16-V, and rK396R were infected at an MOI of 50 at 4°C. Virus-infected cells were incubated for 1 h with polyclonal anti-aMPV/B N protein antibody, followed by staining with TRITC-labeled goat anti-mouse IgG at 4°C for 1 h. Flow cytometric analysis was performed using an LSRII flow cytometer. (**E**) Differences in rLN16-V and rK396R mRNA levels after 1 and 2 h of adsorption. DF-1 cells were infected with rLN16-V and rK396R at an MOI of 50 at 4°C. After three washes with PBS to remove unbound viral particles, the viral mRNA levels were quantified using RT-qPCR. (**F**) Binding affinity of the rLN16-V and rK396R F proteins for αV expressed on the surface of transfected 293T cells. (**G**) Binding affinity of the rLN16-V and rK396R F proteins for β1 expressed on the surface of transfected 293T cells. (**H**) Confocal microscopy analysis of the adsorption capacity of rLN16-V and rK396R to DF-1 cells (50 MOI). Nuclei were counterstained with DAPI. (**I**) The percentage of FITC-positive cells was determined in 200 cells. (**J**) Relative expression levels of F/N protein in nascent viral particles. DF-1 cells were inoculated with rLN16-A and rR396K (MOI = 0.01) at 72 hpi, and the relative ratio of N/F protein was detected. (**K**) The F/N protein intensity ratio was normalized to the control. Data are shown as means ± SD for triplicates from a representative experiment. *P < 0.05; **P < 0.01; ns, no significant difference.

aMPV/B utilizes its F protein to bind to integrin αVβ1 and facilitate viral invasion (47). Sequence analysis of the F gene revealed that residue 396 localized near the αVβ1 binding region of the chicken integrin receptor (44). To verify whether the mutation affects the binding affinity between the virus and αVβ1, DF-1 cells were infected with rLN16-V and rK396R at a dose of 50 MOI, following which the adsorption levels were quantified. The results of flow cytometry showed that the adsorption rate of rLN16-V onto DF-1 cells was 56.4% (Fig. 4A), which was significantly higher than that of rK396R (22.2%) (Fig. 4B). Overexpression assays revealed that rLN16-V adsorbed to 293T cells ectopically expressing ch-αV-HA or ch-β1-HA at rates of 61.1% and 68.7%, respectively, whereas rK396R exhibited adsorption rates of 41.7% and 49.4% (Fig. 4C). Similarly, siRNA-mediated knockdown of chicken integrin αV or β1 in DF-1 cells resulted in adsorption rates of 29.8% and 37.4% for rLN16-V, compared to 14.9% and 18.3% for rK396R (Fig. 4D). The results of RT-qPCR showed that the adsorption of rLN16-V onto DF-1 cells was approximately 4-fold to 8.6-fold higher than that of rK396R (Fig. 4E). The results of protein-cell binding assays also showed that the binding affinity of the rLN16-V F protein to the αV (Fig. 4F) or β1 (Fig. 4G) receptor was markedly higher than that of the rK396R F protein. The results of confocal microscopy showed that the adsorption rate (percentage of FITC-positive cells) of rLN16-V on DF-1 cells was significantly higher than that of the rK396R strain (Fig. 4H and I). Furthermore, to explore whether the K396R mutation affects the incorporation of the F protein into nascent viral particles, the expression levels of the F protein relative to the N protein were quantified in nascent viruses (equal genome copy numbers). The results show that there is no significant difference in expression levels of the F protein relative to the N protein in nascent viruses of rLN16-V and rK396R (Fig. 4J and K), suggesting that the K396R mutation had no impact on integration of the F protein into nascent viral particles. These findings preliminarily suggested that the K396R mutation may decrease the binding affinity between F proteins and the chicken integrin receptor, αVβ1, thus inhibiting the adsorption capacity of aMPV/B on DF-1 cells.

### The structural basis of decreased binding capacity of the F protein to αVβ1 caused by the K396R mutation

**Fig 5:**
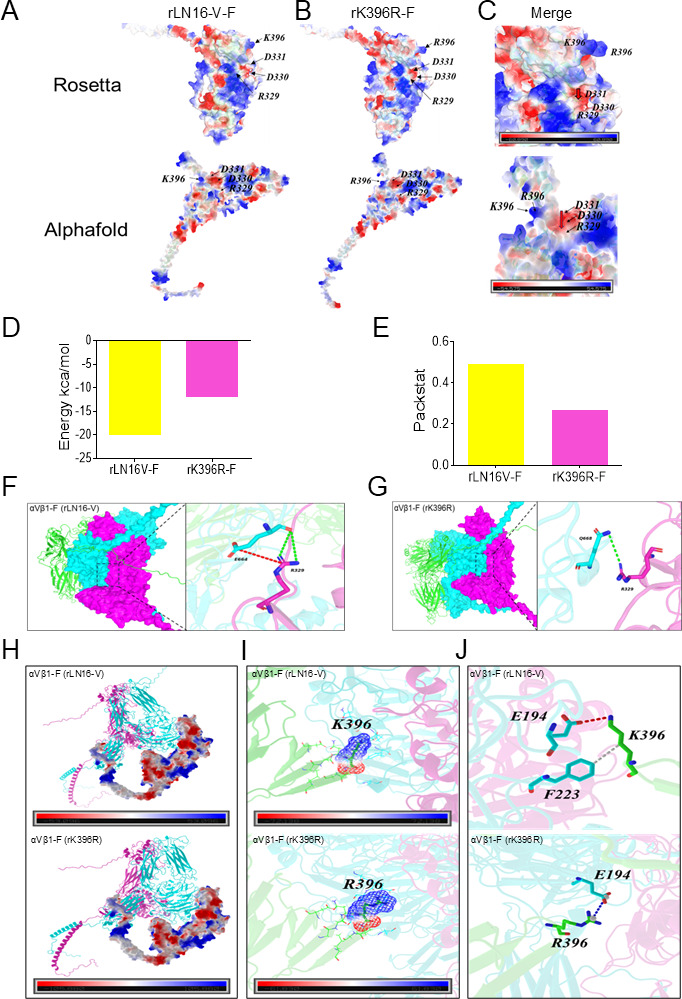
Comparison of the structure of F proteins of rLN16-V and rK396R and molecular docking of F protein with αVβ1. (**A**) Structural prediction of the rLN16-V F protein and surface electrostatic potential of the 329RDD331 binding motif. Using Rosetta and AlphaFold for prediction, respectively. Display of electrostatic potential on the surface of residue K396 and 329RDD331 binding motif. (**B**) Display of electrostatic potential on the surface of residue R396 and 329RDD331 binding motif of the rK396R F protein. The electrostatic potential is calculated and visualized using color. Blue: positive potential; red: negative. (**C**) Change in electrostatic potential on the surface of residue 396 and 329 RDD 331 binding motif of post-overlap F protein (rLN16-V and rK396R). The red arrow shows a decrease in the electrostatic potential of the binding motif 329RDD331 of the rK396R F protein. (**D**) Comparison of the binding free energies of rLN16-V and rK396R F proteins to αVβ1. (**E**) Packstat comparison of the binding of rLN16-V and rK396R F proteins to αVβ1. (**F**) Using Rosetta to analyze the interaction interface between the rLN16-V F protein and αVβ1. Hydrogen and salt-bridge bonds between the key residues of the RDD motif (329R) and the β1 subunit. The αV subunit is in green, the β1 subunit is in cyan, and the F protein is in magenta. The red line represents salt-bridge bonds, and the green lines represent hydrogen bonds. (**G**) Using Rosetta to analyze the interaction surface of the rK396R F protein and αVβ1. Hydrogen and salt-bridge bonds between the key residues of the RDD motif (329R) and the β1 subunit. Green lines represent hydrogen bonds. (**H**) Using Alphafold to analyze the interaction interface between the rLN16-V and rK396R F protein with αVβ1. The αV subunit is magenta, the β1 subunit is cyan, and the F protein is represented in cartoon form. (**I**) The local environment and electrostatic interactions at the 396th amino acid of the F protein. The root mean square deviation (RMSD) for the local environment structure of 396th residue was 0.693 Å (An RMSD of <1.0 Å indicates no significant conformational change). The electrostatic potential calculation results indicate no significant variation in charge distribution within the region of residue 396. (**J**) The side-chain orientation of the 396th residue. rLN16-V F protein residue 329 (R) forms a hydrogen bond with β1 subunit residue E194 and exhibits hydrophobic interactions with β1 subunit residue F223. rK396R F protein residue 329 (R) forms only a salt bridge bond with β1 subunit residue E194. Red shows as a hydrogen bond. Blue shows a salt bridge. White shows a hydrophobic interaction.

To elucidate the potential mechanism underlying the reduced adsorption capacity of rK396R, the structures of the F protein of rLN16-V and rK396R were predicted and analyzed using Rosetta and AlphaFold, respectively. As shown in Fig. 5A and B, the electrostatic potential energy on the contact surfaces of the two F protein (rLN16-V and rK396R) globules was distributed in the form of patches with positive and negative signs, which were calculated and visualized using color (blue, positive potential; red, negative potential). The findings indicated that the K396R mutation significantly decreased (red arrow) the electrostatic potential energy near the αVβ1 binding motif, 329RDD331, of the F protein, increasing the instability in the binding between the F protein and αVβ1 (Fig. 5C). To investigate the effect of the K396R mutation on the interaction of αVβ1 with the F protein, molecular docking methods were used to simulate the binding between αVβ1 and the F protein. The results showed that the binding free energies at the interface between the F protein and αVβ1 of the rLN16-V and rK396R were −19. 9 and −11.9 (Fig. 5D), respectively, and the stacking compactness was 0.492 and 0.269 (Fig. 5E), respectively, indicating that the binding affinity between rK396R F and αVβ1 was lower than that of rLN16-V. Analysis of the interaction on the interface between the rLN16-V F protein (rLN16-V-F) and β1 indicated that the amino acid 664 of the β1 subunit interacts with amino acid 329 of the F protein binding motif and forms two sets of hydrogen and one set of salt bridges (Fig. 5F). In contrast, the amino acid 668 of the β1 receptor subunit interacts with amino acid 329 of rK396R F protein (rK396R-F), forming only one set of hydrogen bonds (Fig. 5G). No interaction sites were detected between the αV subunit and residue 329 of the F protein in either rLN16-V (Table 2) or rK396R (Table 3).

**TABLE 2:** The result of integrin αVβ1 and rLN16-V F protein interaction^***a***^

Interaction	Integrin	F protein	Distance (noH)
Hydrogen bond	αV_M1	Ser143	3.01
Hydrogen bond	αV_R901	D209	3.21
Hydrogen bond	αV_R901	D209	2.74
Salt bridge	αV_R901	D209	4.19
Hydrogen bond	β1_A718	R198	3.00
Hydrogen bond	β1_Q668	M1	2.89
Hydrogen bond	β1_E717	Q195	2.97
Hydrogen bond	β1_K663	R253	2.97
**Hydrogen bond**	**β1_E664**	**R329**	**4.08**
**Hydrogen bond**	**β1_E664**	**R329**	**3.66**
Hydrophobic interaction	β1_F673	L219	3.60
Salt bridge	β1_E724	R205	5.15

^***a***^ Bold font: hydrogen bond between the key residue (329R) of RDD motif in the rLN16-V F protein and the β1 subunit.

**TABLE 3:** The result of αVβ1 and rK396R F protein interaction result^*a*^

Interaction	Integrin	K396 protein	Distance (noH)
Hydrogen bond	β1_V738	Q79	2.71
Hydrogen bond	β1_V738	Q79	3.33
Hydrogen bond	β1_T626	A395	2.87
Hydrogen bond	β1_P736	A76	3.09
**Hydrogen bond**	**β1_Q668**	**R329**	**4.04**
Hydrogen bond	β1_S667	E327	2.75
Hydrogen bond	β1_E664	R285	2.61
Hydrogen bond	β1_T676	R198	3.00
Hydrophobic interaction	β1_T665	V328	3.72
Hydrophobic interaction	β1_F673	L3	3.37

^*a*^ Bold font: hydrogen bond between the key residue (329R) of RDD motif in the rK396R F protein and the β1 subunit.

AlphaFold-based molecular docking revealed a root mean square deviation (RMSD) of 0.693 Å at residue 396, below the 1.0 Å threshold typically associated with significant conformational change, indicating that the mutation does not alter the local environment of residue 396. The electrostatic potential calculations indicate no significant difference in charge distribution between the rLN16-V and rK396R F proteins at residue 396 (Fig. 5H and I). Analysis of side-chain orientation showed that the K396R mutation caused a loss of hydrophobic interactions between the F protein and the β1 subunit residue F223. Furthermore, the hydrogen bond between the F protein 396th residue and the β1 subunit 194th residue changed into a salt bridge (Fig. 5J). Thus, these results suggested that the K396R mutation may be closely related to the decreased binding capacity of the F protein to αVβ1.

### The K396R mutation decreased the binding affinity of the F protein to αVβ1

**Fig 6:**
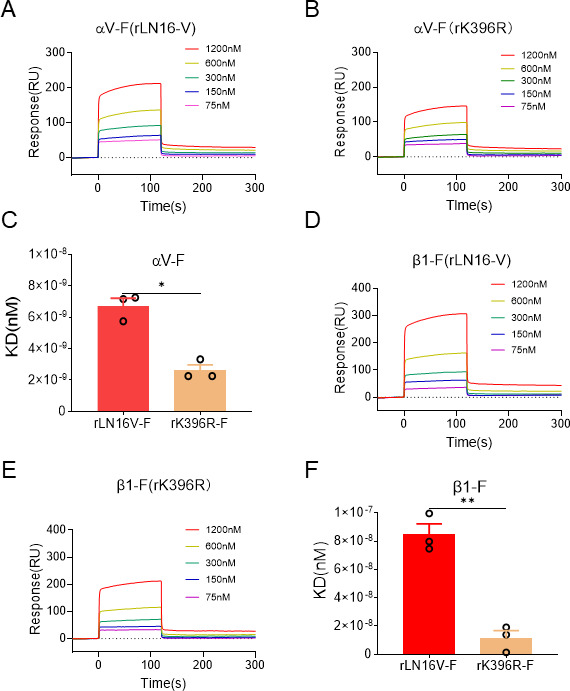
Detecting the binding affinity of rLN16-V and rK396R F proteins for αVβ1. (**A**) Binding kinetics of the αV subunit proteins with rLN16-V F protein. (**B**) Binding kinetics of the αV subunit proteins with the rK396R F protein. The binding affinity of F proteins with the αV was detected using Biacore T200. αV subunit proteins are captured on the chip, and serial dilutions of F proteins were then passed over the chip surface. (**C**) KD values between rLN16-V and rK396R F proteins and αV. (**D**) Binding kinetics of the β1 subunit proteins with rLN16-V F protein. (**E**) Binding kinetics of the β1 subunit proteins with rK396R F protein. β1 subunit proteins were captured on the chip, and serial dilutions of the F proteins were passed over the chip surface. (**F**) KD values between rLN16-V and rK396R F proteins and β1. Data are shown as means ± SD for triplicates from a representative experiment. *P < 0.05; **P < 0.01.

To further analyze the binding of the F proteins (rLN16V-F and rK396R-F) to αVβ1, surface plasmon resonance was used to quantify the binding affinity between various concentrations of the F proteins and αVβ1. At concentrations ranging from 75 nM to 1,200 nM, the response signal values generated by the binding of rLN16V-F to αV (50–212 RU) (Fig. 6A) were higher than those of rK396R-F (38–146 RU) (Fig. 6B). The equilibrium dissociation constant, KD, for the binding of rLN16V-F to αV was calculated to be 6.72 ± 0.7 nM, whereas the KD for rK396R-F binding to αV was only 2.6 ± 0.5 nM (Fig. 6C). Regarding the β1 subunit, at concentrations ranging from 75 nM to 1,200 nM, the response signal values for rLN16V-F binding to β1 (36–306 RU) (Fig. 6D) were significantly higher than those for the rK396R-F (32–212 RU) (Fig. 6E). Additionally, the KD for rLN16V-F binding to β1 was 84.7 ± 21.2 nM, while the KD for rK396R-F binding to β1 was only 15.8 ± 2.41 nM (Fig. 6F), indicating that the binding affinity of rLN16V-F to αVβ1 was significantly higher than that of rK396R-F. These results clearly indicated that the K396R mutation significantly decreased the binding affinity between the F protein of aMPV/B and integrin αVβ1.

## DISCUSSION

aMPV is a major pathogen that threatens global poultry health and causes most acute upper respiratory tract infections (60). Notably, its close relative, hMPV, is a leading cause of acute respiratory illnesses in humans worldwide, highlighting the significant disease burden imposed by metapneumoviruses across species. Following serial passage of aMPV/B SNU21004 virulent strain, Hong SM et al. observed that the ΔG (G-truncation mutant) induced a robust and delayed humoral immune response, with decreased replication ability and virulence to a certain degree (22, 61). However, the specific mechanism underlying its attenuation remains largely unknown. Understanding the molecular basis of viral attenuation is crucial for the rapid development of novel and more effective live vaccines (62). In this study, we demonstrated that the F gene is responsible for the differences in aMPV/B pathogenicity. Specifically, the residue 396K of the F protein was identified as a critical determinant. The K396R mutation in the F protein significantly decreased the binding affinity between the F protein and αVβ1 integrin, a key host cell receptor. This reduction in binding affinity consequently diminished viral replication and pathogenicity in chickens.

Vaccination remains an effective strategy for preventing metapneumovirus infections (63). The commercial vaccines currently available for preventing aMPV in poultry are primarily inactivated and live attenuated vaccines (64, 65). In the field, optimal protection depends on both vaccine type and route of administration. Inactivated vaccine is commonly employed for immunizing breeders and layers due to its high safety and absence of reverse virulence risk (61). However, the efficacy of inactivated vaccines largely depends on the adjuvant involved (66, 67). On commercial poultry farms, mass vaccination with live vaccines via drinking water or spray to achieve herd immunity is widely used as an important tool for disease prevention and biocontainment. However, virulence reversion remains a risk with certain live vaccines (43). In our previous studies, the LN16-A did not revert to a virulent strain after five serial passages in chickens, confirming the absence of reversion risk (41). In this study, we further confirmed that the key virulence 396 site exhibits high genetic stability, as it did not revert to mutation or deletion during both *in vivo* and in *vitro* passage, highlighting its potential application value as a key attenuation site.

Identification of virulence genes is essential for the rapid development of vaccines (68–70). Previous studies have focused on exploring virulence-associated genes or regions of aMPV/A and aMPV/C using reverse genetics (71, 72). The virulence of aMPV/A was significantly decreased by mutating the L gene of the virulent strain, Italy 309/04, to the attenuated strain, rVc (43). In addition, turkeys infected with the rColorado-ΔG strain (deleted G gene) did not develop any clinical signs due to the reduced replication ability of the rColorado-ΔG strain (59). Similarly, hMPV lacking the M2 protein (rhMPVΔM2) was highly debilitated for replication and attenuated compared to the parental rhMPV strain in a hamster model (58). Therefore, the L, G, and M2 genes are currently considered the major virulence genes for aMPV/A, aMPV/C, and hMPV, respectively. Although Miki et al. reported genes that differed among the aMPV/B, the virulent VCO3/60616 strain, and the attenuated VCO3/50 strain (73), the critical gene that determines aMPV/B virulence has not yet been identified. Our previous results confirmed that the genomic differences between the attenuated LN16-A strain and the virulent LN16-V strain were mainly due to six genes: N, P, F, SH, G, and L (41). In this study, we systematically investigated the function of each gene in determining the virulence of aMPV/B using reverse genetics and demonstrated that the F gene is key in determining the virulence of aMPV/B. To the best of our knowledge, this is the first study to systematically identify key virulence genes of aMPV/B.

Single amino acid mutations that attenuate viral pathogenicity are not uncommon and have been described in viruses of the *Pneumovirinae* subfamily, such as hMPV (74), aMPV/A (43), aMPV/C (69), and respiratory syncytial viruses (RSV) (75); the mechanism of attenuation usually involves mutations in the conserved region or in the functional GDNQ motif of the L protein, which reduces polymerase activity, leading to decreased viral replication ability. However, the conserved region and GDNQ motif of LN16-A were not mutated during serial passages, suggesting that attenuation of virulence by decreasing L protein’s polymerase activity is unsuitable for aMPV/B. In this study, the morbidity rate of rK396R infection in SPF chickens was only 10%, demonstrating that the K396R mutation plays a crucial role in attenuating aMPV/B virulence. However, mutation at position 396 alone cannot fully attenuate the aMPV/B virulence, suggesting that other sites affecting viral virulence may also exist. For example, the viral shedding levels induced by rK323E were significantly lower than those induced by rR396K, indicating that the 323rd residue can slightly affect the aMPV/B virulence. Furthermore, studies of other aMPV/B strains have shown that Sun21004(a), lacking the SH and G genes, exhibits attenuated virulence through a mechanism distinct from that of rK396R (22). Therefore, to fully attenuate the virulence of aMPV/B, future studies should also consider the additive effects of the K396R mutation along with other site mutations. Unlike that in hMPV, aMPV (subtype A and C), and RSV, we found that the K396R mutation reduced the adsorption ability and virulence of aMPV/B by decreasing the binding affinity of the F protein to αVβ1. Similar attenuation mechanisms have been observed in other viruses. For example, the K53R substitution of the E_2_ protein in Getah virus alters binding affinity of the heparan sulfate receptor, leading to decreased viral replication in mouse blood and attenuated virulence *in vivo* (76). The D193N mutation in the HA protein of the highly pathogenic avian influenza H5N1 virus reduces the binding affinity of the HA protein for the receptor α2,6-sialic acid, which decreases the replication levels of the virus both *in vivo* and *in vitro* and attenuates its pathogenicity in ferrets (77). The unique attenuation mechanism of LN16-A improves our knowledge regarding the pathogenic mechanisms of viruses of the *pneumoviridae* family and provides a basis for hMPV-related studies. Thus, attenuating the ability of the F protein to bind to the receptor can be used to rationally attenuate pneumoviruses, which might be applied to other animal and human paramyxoviruses for the rational design of live-attenuated vaccines.

The binding of the viral envelope protein to the receptor is a prerequisite for viral infection. Members of the genus *Pneumovirus* primarily bind to the integrin receptor αVβ1 via motifs on F proteins spanning residues 329–331 (RGD, RDD, and RSD) to mediate viral adsorption and invasion (78). Correspondingly, the RDD motif was identified at amino acids 329–331 of the LN16-V and LN16-A F proteins, which were located in the 2a domain of the F1 subunit (170–338 amino acids). Although the RDD-binding motif in the region 2a domain remains unmutated during viral passage, mutations occur in several amino acids close to the RDD-binding motif in the region 2b domain (30). Studies on other viruses have shown that amino acid mutations near the receptor-binding motif may affect the receptor-binding affinity. For instance, the T310K mutation near the E_2_ protein receptor-binding motif of the tick-borne encephalitis virus significantly attenuates the binding affinity of the E_2_ protein to the receptor and diminishes the pathogenicity of the virus in mice (79). The T372A mutation in the SARS-CoV-2 spike protein results in the loss of N-glycosylation at the N370 site, enhancing the affinity of the spike protein for the host cell receptor, ACE2, thereby significantly increasing viral infectivity (80). Similarly, the Q226L mutation in the HA protein of the H7N9 avian influenza virus reduces the stability of the 220 amino acid loop, decreasing the affinity of the HA protein for both human and avian ACE2 receptors, thereby lowering viral replication levels (81). Our results demonstrated that K396R in the region 2b domain is a key amino acid that decreases the binding affinity between the F protein and αVβ1 and reduces the replication ability of aMPV/B. These findings further indicate that the region 2b domain at the C-terminus of the RDD motif of the F1 subunit is a critical region that influences the binding affinity between F and the αVβ1.

The receptor-binding ability of the viral envelope protein depends on its binding free energy, hydrogen bonding, salt bridges, and packing density of the envelope protein (82). A previous study based on the aMPV/C F protein reported that the 2b domain forms the base of a large cavity at the base of the head (29). Although region 2b does not directly contribute to receptor binding, it may alter receptor binding to RDD motifs or attenuate receptor-binding specificity. In this study, the K396R mutation reduced the electrostatic potential of the RDD motif, likely by altering the distribution of non-covalent bonds within the F protein and thereby disrupting the electrostatic equilibrium of neighboring amino acids. In addition, the K396R mutation leads to a reduction in the number of hydrogen and salt-bridge bonds between the RDD motif and αVβ1, which may further form weaker contacts between the F protein and αVβ1. This phenomenon has also been observed during the binding of SARS-CoV-2 (83) to murine leukemia viruses (84). Thus, this may well explain the decreased binding of the F protein to αVβ1 due to the K396R mutation.

In conclusion, the molecular basis of aMPV/B attenuation has been described in this study. The F gene has been identified as the critical aMPV/B virulence gene and the 396th residue of the F protein as the key amino acid that determines pathogenicity. Interestingly, the K396R mutation was shown to reduce the binding affinity of the F protein to its cellular receptor, integrin αVβ1, resulting in significantly impaired viral replication *in vivo*, which is a major factor in viral attenuation. These findings provide fundamental insights into aMPV/B pathogenesis and elucidate the key molecular mechanisms underlying attenuation. Importantly, this study establishes a mechanistic framework that can guide the rational design of novel, more effective live-attenuated vaccines not only for aMPV/B but also for related human pathogens such as hMPV.

## Data Availability

Raw sequencing data have been submitted to the Sequence Read Archive (accession number: PRJNA1417072).

## SUPPLEMENTAL MATERIAL

Supplemental figures mbio.00424-26-s0001.docx Fig. S1 and S2.

ASM does not own the copyrights to Supplemental Material that may be linked to, or accessed through, an article. The authors have granted ASM a non-exclusive, world-wide license to publish the Supplemental Material files. Please contact the corresponding author directly for reuse.
