# Supplementary material for: Mutation of lysine 396 of the fusion protein reduces its binding affinity with integrin αVβ1 and leads to attenuation of avian metapneumovirus subtype B
Source: mBio. 2026 Apr 7;17(5):e00424-26. doi: 10.1128/mbio.00424-26 (PMC13170324; doi:10.1128/mbio.00424-26)
Supplement: Supplemental figures — Fig. S1 and S2. [file mbio.00424-26-s0001.docx]

**
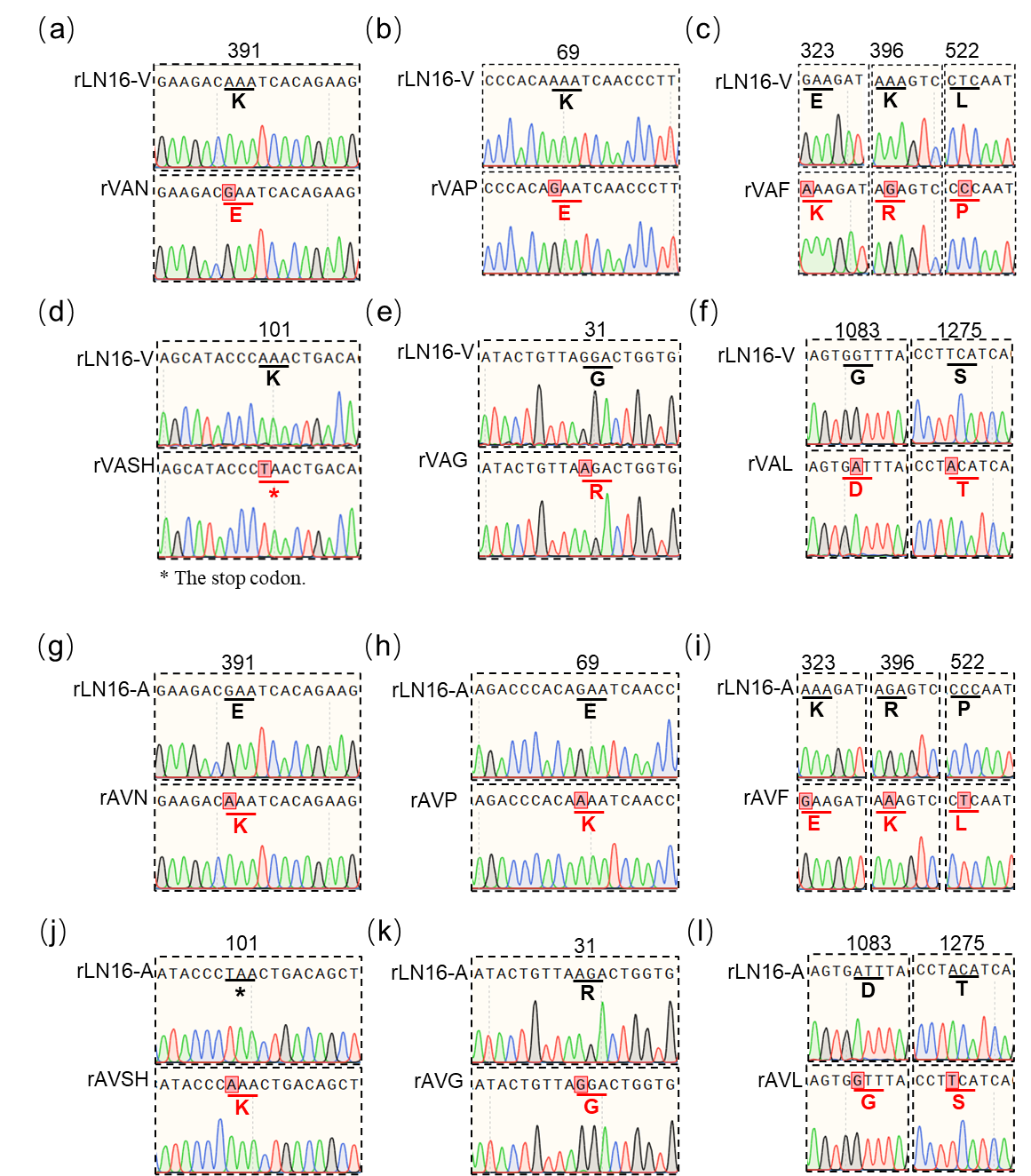
Fig. S1**


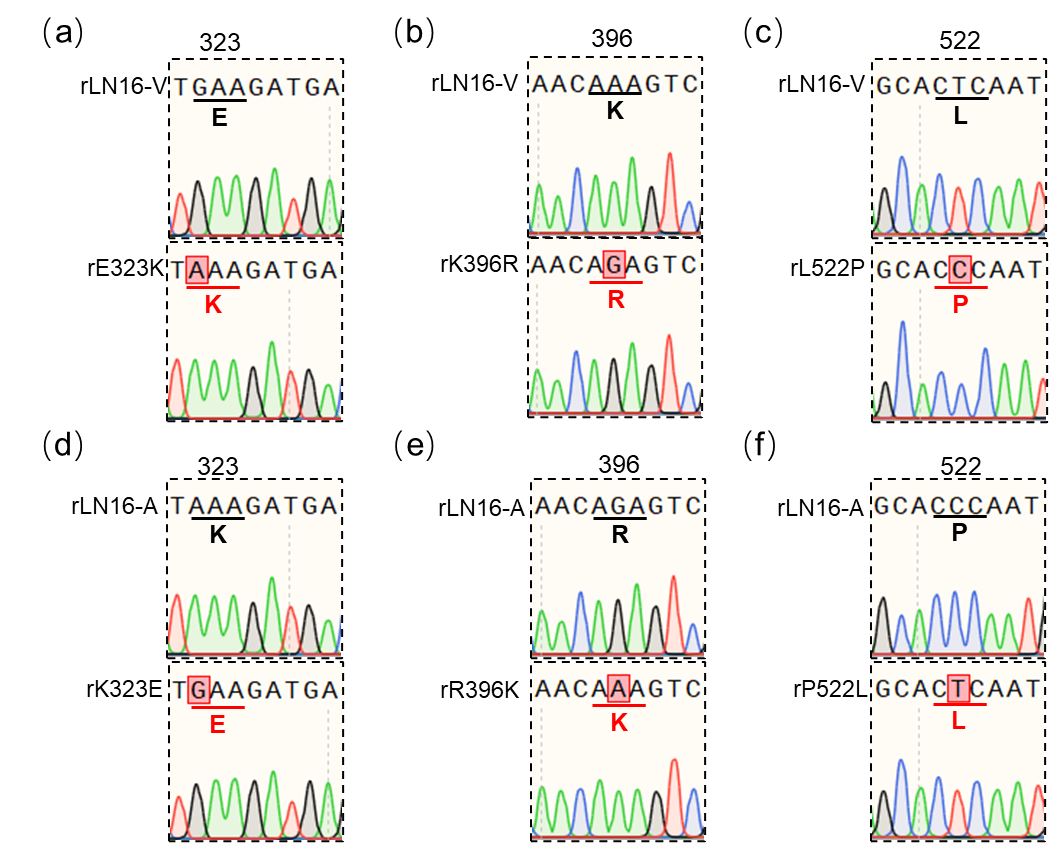
**Fig. S2**

**Figure legends**

**Fig. S1 Identification of gene chimeric viruses.** Molecular sequencing results of gene chimeric viruses, including rLN16-V, rVAN, rVAP, rVAF, rVASH, rVAG, rVAL, rLN16-A, rAVN, rAVP, rAVF, rAVSH, rAVG, and rAVL. (a) Sequencing results of amino acid site 391 in the rVAN strain N gene. (b) Sequencing results of amino acid site 69 in the rVAP strain P gene. (c) Sequencing results of amino acid site 323, 396 and 522 in the rVAF strain F gene. (d) Sequencing results of amino acid site 101 in the rVASH strain SH gene. (e) Sequencing results of amino acid site 31 in the rVAG strain G gene. (f) Sequencing results of amino acid site 1083 and 1275 in the rVAL strain L gene. (g) Sequencing results of amino acid site 391 in the rAVN strain N gene. (h) Sequencing results of amino acid site 69 in the rAVP strain P gene. (i) Sequencing results of amino acid site 323, 396 and 522 in the rAVF strain F gene. (j) Sequencing results of amino acid site 101 in the rAVSH strain SH gene. (k) Sequencing results of amino acid site 31 in the rAVG strain G gene. (l) Sequencing results of amino acid site 1083 and 1275 in the rAVL strain L gene.

**Fig. S2** **Identification of F gene single amino acid mutant viruses.** The results of molecular sequencing of six F gene single amino acid mutant viruses (rE323K, rK396R, and rL522P, rK323E, rR396K and rP522L). (a) Sequencing results of amino acid site 323 in the rE323K strain F gene. (b) Sequencing results of amino acid site 396 in the rK396R strain F gene. (c) Sequencing results of amino acid site 522 in the rL522P strain F gene. (d) Sequencing results of amino acid site 323 in the rK323E strain F gene. (e) Sequencing results of amino acid site 396 in the rR396K strain F gene. (f) Sequencing results of amino acid site 522 in the rP522L strain F gene.
